# Prescribing Medications in Patients with Decompensated Liver Cirrhosis

**DOI:** 10.4061/2011/519526

**Published:** 2011-08-22

**Authors:** Deepak N. Amarapurkar

**Affiliations:** Department of Gastroenterology, Bombay Hospital and Medical Research Centre, New Prabhadevi Road, Prabhadevi, Mumbai 400 025, India

## Abstract

Patients with decompensated liver cirrhosis have various serious complications which require multiple drugs for therapeutic or prophylactic use. Majority of the drugs are primarily metabolized and excreted by hepatobiliary system; hence, liver cell necrosis contributes to impaired drug handling in liver failure while portosystemic shunt can alter drug action in cirrhosis. Hence, in order to decide drug dosing in liver failure, 3 important factors need to be considered (1) pharmacokinetic alterations of drugs, (2) pharmacodynamic alteration of drugs, and (3) increased susceptibility of patients to adverse events particularly hepatotoxicity. Though there is no predictable test which can be used to determine drug dosage in patients with decompensated liver cirrhosis, drugs with first pass metabolism require reduction in oral dosages, for high clearance drugs both loading and maintenance dosages need adjustment, for low clearance drugs maintenance dose needs adjustment, whenever possible measuring drug level in the blood and monitoring of adverse events frequently should be done. No evidence-based guidelines exist for the use of medication in patients' with liver cirrhosis. There are hardly any prospective studies on the safety of drugs in cirrhotic patients. According to the experts opinion, most of the drugs can be used safely in patients with cirrhosis, but drug-induced hepatotoxicity may be poorly tolerated by patients with cirrhosis; hence, potential hepatotoxins should be avoided in patients with liver cirrhosis. Potentially hepatotoxic drugs may be used in patients with liver cirrhosis based on the clinical needs and when there are no alternatives available. Caveat for the prescribing medications in patients with cirrhosis the drug dosing should be individualized depending on a number of factors like nutritional status, renal function, adherence, and drug interaction. Monitoring of the liver function at frequent intervals is highly recommended.

## 1. Introduction

**Figure 1:**
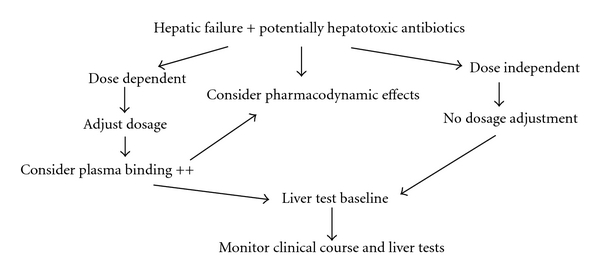
Algorithm for drug dosing in liver failure. Possibly avoids drugs which are metabolized by liver and/or have potential hepatotoxicity.

Liver is a primary site of drug metabolism. Various steps in the drug biotransformation in the liver are entry of the drug in the liver, uptaken by the liver cells, phase I reaction, for example, hydrolysis, hydroxylation, oxidation, reduction, demethylation and phase II reactions conjugation with glycine, glucuronide sulphate, hippurate, and others. These steps are dependent on two factors, hepatic blood flow and metabolic capacity of the liver. In patients with liver cirrhosis impaired drug handling is due to (1) liver cell necrosis, (2) shunting of the blood through porta systemic colaterals, (3) reduction in the concentration of drug-binding proteins, (4) abnormal drug volume distribution, (5) altered drug elimination, (6) altered drug metabolism, (7) altered pharmaco dynamics, (8) associated renal failure, and (9) drug-drug interaction. The impairment of drug metabolism is proportional to the liver dysfunction. Patients with well-compensated cirrhosis and near-normal synthetic function will have a lesser extent of impaired drug metabolism as compared to patients with decompensated cirrhosis with significant synthetic dysfunction and portal hypertension [1, 2]. Though various tests like liver function test, indocyanine green clearance, megaxx, Child Pugh score, and meld score are used for prediction of impaired liver function, still no tests can determine drug dosing in these patients. Drugs with first pass metabolism require reduction in oral dosages, for high clearance drugs both loading and maintenance dosage need adjustment, for clearance drugs maintenance dose needs adjustment (Figure 1). Whenever possible, measuring drug level in the blood and monitoring of adverse events frequently should be done. No evidence-based guidelines exist for the use of medication in patients with liver cirrhosis [3–5]. There are hardly any prospective studies on the safety of drugs in cirrhotic patients. Drug-induced liver injury (DILI) is the commonest cause of drug withdrawal from further development and from the market [6]. Almost 50% of the drugs are associated with some sort of liver injury [7]. Nearly 100 drugs are known to cause fulminant hepatic failure, and 10% of all adverse drug reaction are hepatotoxicity [8, 9]. Approximately 1000 drugs and several herbal remedies have been shown to be causing DILI. Because of this drugs associated with liver toxicity are usually contraindicated in patients with chronic liver disease; still most of the drugs can be used safely in patients with chronic liver disease according to the expert opinion [10–13]. 

According to the experts' opinion most of the drugs can be used safely in patients with cirrhosis, but drug-induced hepatotoxicity may be poorly tolerated by patients with cirrhosis; hence, potential hepatotoxins should be avoided in patients with liver cirrhosis [14]. Potentially hepatotoxic drugs may be used in patients with liver cirrhosis based on the clinical needs and when there are no alternatives available. Caveat for the prescribing medications in patients with cirrhosis the drug dosing should be individualized depending on a number of factors like nutritional status, renal function, adherence, and drug-drug interaction. Monitoring of the liver function at frequent intervals is highly recommended [14, 15]. In spite of these recommendations, monitoring of drug-induced liver injury by alanine transaminase is inconvenient and not followed by both patients and physicians [4]. We as clinicians should educate our patients about the warning signs of drug-induced liver injury like abdominal pain, nausea, and jaundice for stopping the drugs and seeking urgent medical attention.

In this paper after extensive literature search and expert opinions, I will discuss rational use of various drugs in patients with cirrhosis.

## 2. Antibiotic Dosing in Cirrhosis

Liver is an important site of removal of blood bone bacteria. Hepatic destruction of bacteria and reticular endothelial system-related phagocytosis are impaired in patients with cirrhosis. In cirrhotic patients serum bactericidal opsonic activity and neutrophil function are defective. This leads to 5 to 7 fold increase in bacteremia in these patients requiring antibiotics for therapeutic or prophylactic purpose [16]. Extensive literature search was done to identify the antibiotics that need dosage alteration in patients with liver cirrhosis. Macrolide antibiotics like erythromycin, azithromycin, chloramphenicol, lincomycine, and clindamycin which are excreted and detoxified by liver should be used with cautions in these patients. Tetracycline, Isoniazid and Rifampin have prolonged half life in patients with liver cirrhosis. Metronidazole ketocanozole, miconazole, fluconazole, itraconazole, and nitrofurantoin pyrazinamide should be used with caution. Beta-lactum antibiotics can cause leucopenia, while amino glycosides can increase susceptibility to renal failure. Vancomycin can cause increased toxicity in patients with liver failure. Antibiotics which can produce hepatitis or cholestasis should be avoided or used with caution. Tuberculosis was more common in alcoholic and Child class C cirrhosis (Table 1). Antituberculosis therapy (ATT) is associated with hepatotoxicity in 10%. Hepatotoxicity requires withdrawal, modification, and sequential reintroduction to achieve cure of tuberculosis. Using such hepatotoxic drugs in presence of cirrhosis or advanced liver disease is a challenge. Cirrhotic patients with tuberculosis have significantly lower completion of Rifamipicin + Isoniazid based ATT, higher hepatotoxicity, and higher mortality. Recommended ATT in Child class A cirrhosis is the same as a noncirrhotic population but strict followup is required. Pyrazinamide may be avoided. In Child class B Pyrazinamide should be avoided, Isoniazid with rifamipicin may be avoided. Isoniazid or rifamipicin with ethambutol and quinolone can be used for 12 to 18 months. In Child Class C ethambutol, quinolone, and one second line agent may be used for 12 to 18 months [2, 14, 17–19].

Antifungal drugs like Ketocanozole and miconazole though hepatotoxic can be used in patients with cirrhosis but monitor drug concentration in serum is recommended. Metronidazole reduce dose by 50% in patients with severe cirrhosis and/or associated renal insufficiency. There is no information of safe use of nitrofurntoin, chloramaphenicol, sodium fusidate and pyrazinamide but they are poentially toxic hence avoid their use in liver disease [14] (Tables 2 and 3). 

## 3. Sedation, Anesthesia and Analgesia in Patients with Liver Cirrhosis

Endoscopic procedures are often necessary in patients with cirrhosis who may need sedation or short anesthesia. Benzodiazepines like midazolam administered in single dose have minimal impact in patients with compensated cirrhosis. Benzodiazepines can be cautiously used in decompensated cirrhosis. Flumazenil can be used to reverse the effect of benzodiazepine. Fentanyl (opioid) elimination is near normal in cirrhotics and can be used for sedation. Patients with opioid toxicity can be treated with nalaxone; propofol is preferred to benzodiazepines or opioids for endoscopic sedation for patients with decompensated cirrhosis due to its short half life and lower risk of inducing encephalopathy. In patients without extrahepatic high risk, the gastroenterologist directed propofol is safe. The adverse effects of propofol are hypotension, tachycardia, hypoventilation, and prolongation of QT interval [1, 20–22].

## 4. Anesthetic Agents

General Anesthesia can reduce the hepatic blood flow resulting into decompensation. Volatile agents and halothane should be avoided. The new agents like isoflurane, desflurane are not significantly metabolized by the liver; hence, are safe. Combination of agents like fentanyl may greatly reduce the need of anesthetic agents. Propofol is also a good agent for combination anesthesia [14, 23].

## 5. Analgesics

Pain management in cirrhosis is a challenging task as use of analgesic agents is associated with severe complications like gastrointestinal bleeding, hepatic encephalopathy, hepatorenal syndrome, and mortality. Nonsteroid anti-inflammatory agents are contraindicated as they can induce GI bleed and renal failure. Opioid analgesic should be used with caution as it can precipitate encephalopathy. Acetaminophen at a dose less than 2 gm/day is a reasonably safe option. Patients with cirrhosis having visceral or musculoskeletal pain should be treated with acetaminophen less than 2-3 gms/day [24, 25]. In case of inadequate pain relief, tramadol 25 mg every 8 hours can be used. For intractable pain hydromorphone orally or fentanyl topical patch can be used. Combination of these drugs with tramadol should not be done. Neuropathic pain can be treated with nortriptyline, desipramine, and gabapentin, pregabalin with or without acetaminophen. Analgesic choice in patients with cirrhosis should be individualized depending on etiology of cirrhosis, nutritional status, adherence, renal function, liver transplant candidacy, and drug-drug interaction [14, 20, 22].

## 6. Anticonvulsants

Phenytoin, Carbamazapin, and valproate can be hepatotoxic. All the drugs can be used in patients with decompensated liver disease with caution. The newer anticonvulsants like lamotrigine, topiramate also need lowering of the dosage in cirrhotic patients. Antidepressant, (selective serotonin reuptake inhibitors) like fluvoxamine, paroxetine, and fluoxetine need dose modification in patients with cirrhosis [14].

Antiemetic metoclopramide and ondansetron require significant dose reduction in patients with cirrhosis. As antiulcer agents proton pump inhibitors are preferred over H2 receptor blockers but they still need half the dosage [14]. 

## 7. Cardiovascular Drug Therapy

Patients of nonalcoholic steatosis-related cirrhosis have increased incidence of dyslipidemia, hypertension, and coronary artery disease. Drugs like labatolol and methyldopa can cause severe hepatotoxicity and need frequent monitoring and should be used only when there are no other choices. Captopril, Amiodarone, and ticlopidine can cause hepatotoxicity and should be used with caution. The details of dose adjustments on alpha blockers, ACE inhibitors, angiotensin II receptor antagonist, and other drugs used in cardiovascular diseases have been reviewed in the recent past [26]. Satins appear to be remarkably safe in patients with liver cirrhosis [27].

Drug-induced liver injury has been reported almost in 50% drugs in the physicians' desk reference. More than 100 drugs are incriminated in causing fulminant hepatic failure. The drugs that have been mentioned to be contraindicated in the patients with liver disease are methotrexate, niacin, Naltrexone, Metformin, Novastatin, Felbamate, Ticklopidine, Clonazipam, Gemfibrizil, valproic acid, and estrogens in the physicians' desk reference, but some of them are used in clinical practice under strict supervision. Metformin can be used in patients with liver cirrhosis without renal insufficiency. Other antidiabetics like second-generation sulfonylurea like Glipizide, Glimepride may be the drug of choice in patients with liver cirrhosis. Thiazolidinediones can cause drug hepatitis but can be used in reduced dosage with strict monitoring [14, 15].

In conclusion prescribing medicines in patients with liver disease is a challenging task. There are no clear tests which can identify altered drug metabolism in these patients. Medications should be individualized depending upon various factors. Surveillance using liver enzymes though recommended routinely the use of INH can lead to acute liver failure despite the surveillance. The enhanced Nephrotoxicity of radio contrast agents Aminoglycosides and NSAID may be a more frequent and dangerous challenge than hepatotoxicity.

## Figures and Tables

**Table 1:** Antibiotics to be avoided in liver disease.

Chloramphenicol—higher risk of bone marrow suppression (markedly increased half life)
Erythromycin estolate: causes cholestasis
Tetracycline—dose related hepatotoxicity
Antituberculous therapy in combinations, pyrazinamide
Griseofulvin—contraindicated
Nalidixic acid
Nitrofurantoin prolonged use

**Table 2:** Antibiotics which need to be used with extra caution in patients with liver failure.

Piperacillin	Nalidixic acid	Azithromycin
Ceftazidime	Pefloxacin	Tetracycline
Ceftriaxone	Gatifloxacin	Cotrimoxazole + Trimethoprim
Cefoperazone	Erythromycin	Metronidazole
Cefoperazone + Sulbactam Cefetamet	Roxithromycin	Ketoconazole & other fluconozoles

**Table 3:** Antibiotics causing hepatotoxicity.

Hepatocellular injury	Cholestatic injury	Fulminant hepatic failure
Chloramphenicol, Clindamycin	Cephalosporins, Erythromycin	Sulfonamides, Trimethoprim—Sulfomethoxazole
Penicillin G, Amoxicillin	Penicillin G, Oxacillin, Cloxacillin	Ketoconazole, PAS, Trovafloxacin
Trimethoprim—Sulfomethoxazole	Floxacillin, Augmentin, Clarithromycin	
Amphotericin, Hydroxystilbamidine	Nitrofurantoin, Trimethoprim—Sulfomethoxazole	
Ketoconazole, Itraconazole	5-fluorocytosine, Griseofulvin	
INH, Trovafloxacin, Oxacillin	Trovafloxacin, Thiabendazole	
